# Mortality due to Multidrug-Resistant Gram-Negative Bacteremia in an Endemic Region: No Better than a Toss of a Coin

**DOI:** 10.3390/microorganisms11071711

**Published:** 2023-06-30

**Authors:** Olga Tsachouridou, Dimitrios Pilalas, Sideris Nanoudis, Athanasios Antoniou, Isidora Bakaimi, Theofilos Chrysanthidis, Konstantinos Markakis, Angeliki Kassomenaki, Paraskevi Mantzana, Efthymia Protonotariou, Lemonia Skoura, Symeon Metallidis

**Affiliations:** 1 Infectious Diseases Unit, 1st Internal Medicine Department, AHEPA University Hospital, School of Medicine, Aristotle University of Thessaloniki, 55436 Thessaloniki, Greecemetallidissimeon@yahoo.gr (S.M.); 2 Department of Microbiology, AHEPA University Hospital, School of Medicine, Aristotle University of Thessaloniki, 55436 Thessaloniki, Greecemollyskoura@gmail.com (L.S.)

**Keywords:** Gram-negative bacilli bacteremia, multidrug resistance, 28-day mortality, procalcitonin, rapid molecular diagnostics

## Abstract

The incidence of multidrug-resistant (MDR) bloodstream infections (BSIs) is associated with high morbidity and mortality. Little evidence exists regarding the epidemiology of BSIs and the use of appropriate empirical antimicrobial therapy in endemic regions. Novel diagnostic tests (RDTs) may facilitate and improve patient management. Data were assessed from patients with MDR Gram-negative bacteremia at a university tertiary hospital over a 12-month period. In total, 157 episodes of MDR Gram-negative BSI were included in the study. The overall mortality rate was 50.3%. Rapid molecular diagnostic tests were used in 94% of BSI episodes. In univariate analysis, age (OR 1.05 (95% CI 1.03, 1.08) *p* < 0.001), Charlson Comorbidity Index (OR 1.51 (95% CI 1.25, 1.83) *p* < 0.001), procalcitonin ≥ 1(OR 3.67 (CI 95% 1.73, 7.79) *p* < 0.001), and monotherapy with tigecycline (OR 3.64 (95% CI 1.13, 11.73) *p* = 0.030) were the only factors associated with increased overall mortality. Surprisingly, time to appropriate antimicrobial treatment had no impact on mortality. MDR pathogen isolation, other than *Klebsiella pneumoniae* and *Acinetobacter baumanii*, was associated with decreased mortality (OR 0.35 (95% CI 0.16, 0.79) *p* = 0.011). In multivariate analysis, the only significant factor for mortality was procalcitonin ≥ 1 (OR 2.84 (95% CI 1.13, 7.11) *p* = 0.025). In conclusion, in an endemic area, mortality rates in MDR BSI remain notable. High procalcitonin was the only variable that predicted death. The use of rapid diagnostics did not improve mortality rate.

## 1. Introduction

Blood stream infections (BSIs) caused by multidrug-resistant (MDR) Gram-negative bacteria (GNB) are a healthcare-associated issue causing the highest burden in quality of life and are also associated with poor clinical outcomes [1,2].

They are relatively frequent among intensive care unit (ICU) patients and are associated with significant mortality [3,4,5,6]. Recently, worrisome increases in antimicrobial resistance have been highlighted globally [7,8,9]. Antimicrobial resistance is associated with delays to adequate antimicrobial therapy and increased mortality [10,11]. It leads to exacerbation in the use of broad-spectrum antimicrobial agents that in turn enhances the rise of more resistant pathogens [12,13]. The presence and spread of MDR GNB dramatically restricts treatment options for such nosocomial infections [14], whereas the pipeline of novel antimicrobial agents is slow, and various compounds, including tigecycline, have not always proved to be promising [15,16]. Monotherapy with formerly abandoned antibiotics, such as polymyxins, is another option [17]. However, beside resistance, toxicity issues may arise [18].

Over the past years, *Acinetobacter baumannii* and *Klebsiella pneumoniae* have emerged as severe nosocomial pathogens due to their extensively resistant antimicrobial profile in blood cultures in endemic regions of Europe [14,19,20]. The extent of resistance may vary for each isolate; therefore, distinct definitions have been formed. Bacteria expressing in vitro resistance to three or more antimicrobial categories are referred to as multidrug-resistant organisms [21].

The combined use of antimicrobial agents has been applied in the management of infectious diseases, lately garnering more attention by clinicians due to the aforementioned reasons in Europe [22,23]; however, more importantly in the case of MDR Gram-negatives, it is expected to provide a probable antimicrobial synergy [24] and improve survival [25]. However, empirical treatment remains inadequate in a significant proportion of Greek hospitals and rather high mortality is recorded in both ICU and medical ward settings [26]. Moreover, the local epidemiology and the limited feasibility for isolation of patients with MDR infections in Greek hospitals have a negative impact on efforts for effective infection control programs [20].

Although BSIs occur less often than other nosocomial infections [27], the isolation of a pathogen in blood is solid evidence of severe infection [28]. Moreover, during the COVID-19 pandemic, a substantial rise in hospital onset of MDR infections has been reported in some Greek regions [29], compared to previous years. Although this observation could be attributed to multiple reasons, such as the prolonged length of stay of such patients, rapid burst in the number of critically ill individuals, the use of external devices, and the excessive consumption of in-hospital antimicrobials.

Consequently, the treatment of MDR GNB infections remains a challenge. Since an effective treatment should be administered promptly, antimicrobial resistance almost invariably leads to inadequate empirical treatment, with possible negative consequences [30]. To optimize antimicrobial treatment aside from the knowledge of local epidemiology, medical history of patients, and the risk of MDR GNB infection, the improvement and implementation of rapid molecular resistance typing techniques will assist the selection of the proper antibiotic. Finally, a rapid diagnosis will improve targeted therapy through the prompt initiation of adequate therapy and the de-escalation of antimicrobials when susceptibility results are available [30].

The aim of this study was to investigate the impact of MDR GNB bloodstream infection on the primary outcome of 28-day mortality of all causes. In addition, we tried to assess other risk factors for mortality in patients with GNB in an endemic area to the settings of both ICUs and medical wards during the last year of the COVID-19 pandemic.

## 2. Materials and Methods

### 2.1. Study Design, Setting, and Selection Criteria

This was a retrospective study, from 1 January 2022 until 31 January 2023, which was carried out at AHEPA University Hospital, a 700-bed institution with 4 ICUs, as well as surgical and internal medicine departments, that serve patients from a large region of Northern Greece. Microbiology data were retrieved from the laboratory database, while medical history and epidemiological and treatment data were reviewed from the patients’ electronic health records. The Institutional Medical Scientific Board approved this study (Scientific Council, Institutional Review Board AHEPA University Hospital, Ref 018/06.02.2023).

The patients included in the study fulfilled the following criteria: adult ≥ 18 years old and first BSI episode due to a MDR GNB pathogen. We excluded individuals with Gram-positive, Gram-negative (other than MDR), or fungal BSI. Polymicrobial infections, defined as more than one pathogen isolated in a set of blood cultures, were also included in the study, even though such results may have influenced the provider’s decision on antimicrobial modification. Patients who died or were discharged prior to culture results were also excluded from the analysis.

No informed consent was required, as we handled the individual patient data anonymously. We report our results based on the Statement on Strengthening the Reporting of Observational Studies in Epidemiology [31]. 

### 2.2. Variables

Variables of interest on admission were: age, sex, co-morbidities (coronary heart disease, peripheral vascular disease, dementia, peptic ulcer, liver disease, diabetes mellitus, hemiplegia, chronic kidney disease, solid tumor, immunosuppression, AIDS or COVID-19 infection), Charlson Comorbidity Index [32], ICU admission during hospitalization, length of in-hospital stay, medical or surgical reason of hospitalization, infectious disease status, and presence of immunosuppression or COVID-19 co-infection. We documented the day of the BSI event, its timing (<48 h or ≥48 h from admission), the source of bacteremia, the type of pathogen isolated, and whether the infection was monomicrobial or multimicrobial. We also recorded baseline creatinine and procalcitonin on the sample date of blood culture, antimicrobial resistance patterns in GNB blood culture isolates, use of rapid molecular diagnostic test (BIO-FIRE, Salt Lake City, UT, USA, FilmArray^OR^) that was currently available in our hospital, antimicrobials used to treat the infection (empiric and targeted), appropriateness of empiric antibiotics as well as source control, time to targeted treatment, all-cause mortality within 28 days of BSI episode, as long as actions taken upon receipt of susceptibility results (antibiotic de-escalation, adequate targeted treatment).

### 2.3. Definitions

We defined MDR GNB BSI as a positive blood culture from a MDR GNB, and the patient presented clinical and laboratory indices of infection. Index day was the date of collecting the first positive blood culture (index culture) that recovered a multidrug Gram-negative isolate. We examined all patients’ relevant MDR GNB BSI episodes for the purposes of the study. Bacteremia was assessed as primary or secondary based on whether the blood infection occurred directly or spread from another site of the body. Bacterial identification and antimicrobial susceptibility testing were performed using the automated system VITEK2 (bioMérieux, Marcy l’Etoile, France). Susceptibility testing results were interpreted according to the EUCAST breakpoints v 12.0 (2022) [33]. All isolates were tested phenotypically for the detection of metallo-β-lactamases (MBL), Klebsiella pneumoniae carbapenemase (KPC), or both categories, using the meropenem disc test [34]. The combined meropenem disc test is a test where four meropenem discs are used with and without carbapenemase inhibitors (EDTA and phenylboronic acid). First, a 0.5 McFarland bacterial suspension of the tested bacteria is inoculated onto a Mueller–Hinton agar plate. Then, four meropenem discs are placed on the surface of the agar: the first disc is left without inhibitors; 10 μL of EDTA 0.1 M is added on the second; 20 μL (20 g/L) phenylboronic acid is added on the third disc; and on the fourth disc, both inhibitors are applied. The evaluation of the results is performed after 18–24 h of incubation at 37 °C. The diameter of the growth-inhibitory zone around the meropenem discs with EDTA and phenylboronic acid are compared with that around the plain meropenem discs as follows. No inhibition zone around the plain first disc or an inhibition zone with a diameter of <22 mm for Enterobacterales or <14 mm for *P. aeruginosa* is indicative of carbapenem resistance. An inhibition zone around the second and the fourth disc with a diameter ≥5 mm than that of the first disc indicates MBL production. An inhibition zone around the third and the fourth disc with a diameter ≥5 mm than that of the first disc indicates KPC production. An inhibition halo around the second and third disc with a diameter ≥5 mm than that of the first disc and an even larger halo around the fourth disc indicates simultaneous MBL and KPC production.

Further assessment was the modification of therapy within 24 h of the reported susceptibilities. Modification was defined as either escalation of therapy to broader coverage or a de-escalation to a targeted agent based on the results of rapid diagnostic tests. Antibiotic therapy was categorized on empirical and targeted treatment, either as monotherapy either as a combination of antimicrobials. Adequate empirical antibiotic therapy was defined as the initiation of at least one antimicrobial agent to which the isolate from blood culture was susceptible within 24 h after the blood sample drawn; this definition was adapted from previous studies [35].

Of note, many patients were not initially on broad-spectrum antimicrobials and in many others targeted therapy did not include a narrow-spectrum antibiotic. All outcomes measuring time were measured from the time of blood culture draw based on results reported by the microbiology department. Other variables included time to targeted therapy, modification within 24 h of susceptibility results, modification of treatment from empiric combination, hospital length of stay, and mortality on day 28. Time to targeted therapy was defined as the time from the culture draw to the time of escalation or de-escalation to an antibiotic with in vitro activity against the isolated pathogen.

### 2.4. Outcome

The main study outcome was overall 28-day all-cause mortality after the withdrawal of the positive blood culture and the isolation of MDR Gram-negative isolates. Further sub-analysis was performed separately based on the isolate of BSIs with regards to mortality and possible risk factors for death.

### 2.5. Statistical Analysis

Continuous variables were presented as the mean and standard deviation (age, CCI index) or median and interquartile range (IQR), such as hospital or ICU days, whereas categorical variables were presented as frequencies and percentages (ward setting, pathogen isolated, type of bacteremia, Filmarray use, number of active drugs, COVID-19 co-infection). Logistic regression analysis was used to examine the association of different parameters with 28-day mortality. Parameters with a *p*-value < 0.2 in the univariate regression model as well as clinically significant parameters were entered into a multivariable regression model. The same analysis was run separately in patients with *A. baumanii* and in patients with *K. pneumoniae*. Statistical analysis was performed using STATA 17.0 software and the significance level was set at α = 0.05. We performed multivariable logistic regression analysis to evaluate risk factors for mortality in patients with MDR BSI.

## 3. Results

### 3.1. Baseline Characteristics of Patients with MDR GNB BSI Episodes

A total of 157 MDR GNB bloodstream infection episodes were included in the study. Demographical, clinical, and laboratory data are summarized in Table 1. Eighty-three patients were males (52.8%) and the mean age of the cohort was 67.63 years old. Nearly 58% of the episodes referred to patients from medical wards and 42% to ICU patients. The vast majority of the participants had normal renal function upon admission, a mean Charlson Comorbidity Index below 4, and median length of hospital stay of 30.5 days. In 56% of episodes, the patient had a procalcitonin measure of ≥1 ng/mL. Notably, 47% of patients never received an adequate antimicrobial therapy, while 31% received adequate treatment within 24 h of the blood sample draw. Nearly half of the isolates referred to infection from *A. baumanii*, followed by *K. pneumoniae* (25%) and 25% other MDR GNB (*Pseudomonas aeruginosa, Proteus mirabilis, Enterobacter cloacae, Providencia stuartii*). Approximately 60% of BSI episodes were primary bacteremias and 85.7% of bacteremias were monomicrobial. FilmArray was used in the vast majority of cases (152/157). Empirical treatment included at least one active agent against GNB in less than one-third of the episodes (31.06%), while 69% had no active antimicrobial in their initial empirical combination. The proportion of adequate treatment improved to 50.3% upon susceptibility results. Intervention with regards to the discontinuation of unnecessary antimicrobial agents was reported in 36% of cases, while 68% of patients continued to receive an extended combination.

Thirty-four percent of MDR GNB bacteremias were reported among COVID-19 patients who were currently hospitalized during the study period, which highlights the impact of the pandemic on nosocomial morbidity even at its ending period.

MBL production was detected with different methods in 51 cases (31.68%), while KPC was reported in 37 cases (23%). Colistin was part of the empirical treatment in 14% of BSI episodes, tigecycline in 11%, and combination of both agents in 11% as well. Tigecycline was also part of combined empirical treatment with other novel antimicrobials such as ceftazidime/avibactam in lower rates (Table 1).

Overall mortality reached 50.3% in this cohort during the study period. More ICU patients with MDR Gram-negative bloodstream infection died (37 ICU patients dead vs. 29 ICU patients alive) in 28 days. This may be attributed to the fact that patients in the ICU are critically ill, more frequently septic, and could inevitably die regardless of antimicrobial treatment. Patients who died had also higher CCI score, higher procalcitonin count, more impaired renal function at admission, and received less frequently adequate empirical or targeted treatment compared with those who survived until day 28, as illustrated in Table 1.

### 3.2. Univariate and Multivariate Analysis for 28-Day Mortality

In univariate analysis for all-cause 28-day mortality (Table 2), we observed that for each one-year increase in age, the odds of death increased by 5% (OR 1.05 (95% CI 1.03, 1.08) *p* < 0.001). More significantly, the odds of death increased by 51% for each one-unit increase in Charlson Comorbidity Index (OR 1.51 (95% CI 1.25, 1.83) *p* < 0.001). The hospitalization setting (internal medicine ward, surgical ward, ICU) did not seem to affect the primary outcome of interest. Mortality from MRD GNB bacteremia was not also associated with days of hospitalization, even in the ICU, a finding that is quite concerning for patients with less severe morbidity. The use of molecular rapid diagnostic tests was quite frequent in our cohort. However, this tool did not offer any positive impact on mortality in MDR GNB in an endemic environment, which highlights the need for further investigation of the factors that will improve survival and in-hospital morbidity in these patients.

Monomicrobial infections were not associated with lower mortality rates from MDR Gram-negative bacteria, which is also an important finding. Our cohort included a non- neglectable proportion of multi-microbial bacteremias, reaching 13% of the total episodes during the study period of interest. Literature remains ambiguous concerning polymicrobial vs. monomicrobial multidrug Gram-negative bacteremias.

Patients with PCT ≥ 1 ng/mL at the time of blood sample draw had 3.7 times higher odds of death than the patients with PCT < 1 ng/mL (OR 3.67 (CI 95% 1.73, 7.79) *p* < 0.001), which firmly supports the use of procalcitonin in severely affected patients. Patients with BSI isolates other than *A. baumanii* or *K. pneumoniae* had 65% lower odds of death compared to patients with *K. pneumoniae* (OR 0.35 (95% CI 0.16, 0.79) *p* = 0.011). Administration of tigecycline as empiric monotherapy in an endemic area was found to have a negative impact on patients, since they had 3.6 times higher odds of death compared to those who did not receive tigecycline (OR 3.64 (95% CI 1.13, 11.73) *p* = 0.030). Patients with ≥2 active antibiotic agents in targeted treatment had 65% lower odds of death compared to patients with no adequate targeted treatment (Table 2).

The different mechanisms of antimicrobial resistance did not have different impacts on 28-day mortality among bacteremic patients with multidrug-resistant Gram-negative bacteria. This finding could probably be explained in the setting of endemicity of MDR Gram-negative pathogens in the blood of hospitalized patients. Overall mortality remains high, and more prevention measures and treatment management protocols need to be evaluated and implemented to improve outcomes.

Other probable risk factors for the outcome of interest were the number of active drug agents in both empirical and targeted treatment. Even though it would be expected that more effective antimicrobials in a prescribed regimen would be life-saving for hospitalized patients, our data did not confirm an association with lower 28-day mortality. Furthermore, the modification of treatment post-susceptibility results and discontinuation of unnecessary agents had no impact on the outcome. The continued administration of redundant agents may be associated with toxicity and adverse events, such as *Clostridioides difficile* colitis, thus increasing the morbidity and mortality risk, but this was not illustrated here.

In multivariate analysis (Table 3), only patients with PCT count ≥ 1 had 2.8 times higher odds of death than patients with PCT < 1, adjusted for all the other variables in the model (OR 2.84 (95% CI 1.13, 7.11) *p* = 0.025).

### 3.3. Patients with BSI from MDR A. baumanii

When we performed univariate analysis among patients with MDR *A. baumanii* bacteremia, we observed that for each one-year increase in age, the odds of death increase by 9% (OR 1.09 (95% CI 1.04, 1.14) *p* < 0.001). With regards to CCI score, for each one-unit increase in Charlson Comorbidity Index, the odds of death increase by 78% (OR 1.78 (95% CI 1.29, 2.46) *p* < 0.001). Similar to the whole cohort of BSIs, patients with PCT ≥ 1 have 3.5 times higher odds for mortality than the patients with PCT < 1 (OR 3.45 (95% CI 1.08, 1.03) *p* = 0.037). The co-administration of colistin plus tigecycline in empirical treatment led to a decrease in odds of death by 79% (OR 0.21 (0.05, 0.87) *p* = 0.032).

### 3.4. Patients with BSI from MDR K. pneumoniae

Accordingly, when studying separately patients with *K. pneumoniae* using univariate analysis, we observed that patients having PCT ≥ 1 have 3.7 times higher odds of death than patients with PCT < 1. Additionally, patients with more than one active antibiotic in empirical treatment have 84% lower odds of death compared to patients with no active antibiotic agents in their initial antibiotic regimen. As could have been expected, patients with time to adequate antimicrobial therapy of greater than 24 h were assessed to have 7.5 higher odds of death than those with time to adequate antimicrobial therapy lower than 24 h, highlighting the fundamental principal of infection control that time to appropriate treatment is of great significance for an optimal outcome.

## 4. Discussion

The prevalence of bloodstream infections attributed to MDR GNB is currently rising with negative impact on morbidity and mortality. Epidemiology data on the prevalence and circulating antimicrobial resistance patterns of GNB BSI isolates from hospitalized patients, as well as the identification of risk factors for harboring MDR GNB infections, may facilitate patient care [35]. Our aim was to describe mortality in bacteremic patients from MDR Gram-negative bacteria in a tertiary hospital in Thessaloniki, Greece, a region endemic for multidrug and difficult-to-treat Gram-negative hospital infections.

Our study revealed a somewhat high case mortality rate of 50.3% among patients with MDR GNB bloodstream infections, which was much higher than previous published studies [36] of hospitalized patients. Mortality rate was assessed as lower (41.6%) in neutropenic patients [37], pediatric (21.4%) patients [38], or strictly among ICU (45%) patients [39,40], according to other authors. Our population differs from the above in terms of heterogeneity, with regards to medical history, comorbidity status, and hospital ward origin (both ICU and non-ICU participants). This fact could have accounted for such differences in mortality rates. Several studies pointed out that ICU admission is a risk factor for poorest survival [41], a finding thus not confirmed in our data, which show high mortality rates in both hospitalization settings. However, we should mention that in non-endemic countries for MDR GNB bacteria, even more dramatic mortality rates have been reported in the ICU setting [42]. However, we should underline that ICU patients are critically ill and may suffer from sepsis, and thus die irrespective of antimicrobial treatment.

Patients with MDR GNB infections are more likely to receive inadequate empirical treatment [43] leading to poorer outcomes, such as increased mortality and prolonged hospitalization [44]. In a large multicenter study of ICU patients run in 52 countries, adequate antimicrobial therapy was received by 51.5% within 24 h of blood culture draw [40]. Additionally, antimicrobial resistance was associated with delay to adequate antimicrobial treatment [40]. In our study, inadequate empirical treatment was not associated with higher mortality, in contrast with previous studies [36,45]. This could be probably attributed to large proportions of both inadequate empirical and targeted treatment options in this study, along with being investigated in an endemic setting for MDR GNB.

Ten years after a similar multicenter cohort study of critically ill patients with BSIs [3], comparable delay to adequate antimicrobial therapy was reported by others [40], underlining the need for implementation of integrated protocols and infection control programs to better predict antimicrobial resistance and source control. Antimicrobial resistance was associated with a delay in the administration of effective treatment. Delayed adequate antimicrobial therapy was not associated with 28-day mortality. Such observation may be impacted by several confounders and should be interpreted with caution, as also underlined by other larger studies [40]. Limited data report adequate antibiotic therapy in ICU patients with MDR bacteria. An Italian study reported 61% inadequate treatment for MDR infections [46], which is, however, more consistent with our rates, in both hospital settings.

Indeed, many observational studies consider the possible relation between all-cause mortality and time to appropriate antimicrobial therapy as complicated and difficult to be clarified [47]. On the one hand, the clinical assessment of severity of infection may guide the prompt administration of broad-spectrum antimicrobials to patients at higher risk of death and thus to confound the results of such studies.

The findings of this study do not relegate early adequate antimicrobial therapy recommendations for patients with severe infections. Indeed, while avoidance of antibiotic overuse and its associated adverse events [48], primary adequate antimicrobial treatment remains an intervention of great significance for nosocomial BSIs [49]. Integrated infection control and antibiotic stewardship programs may facilitate clinical management providing advice and recommendations on antibiotic selection, mode, and dosing of administration, as well as the schedule for monitoring clinical and laboratory course of treatment [49].

Comorbidities have been assessed to play a significant role in poorer outcomes [36,37] and high mortality rates. In our study, higher Charlson Comorbidity Index was risk factor for high mortality in univariate analysis, which is in line with other authors [36,41]. Remarkably, CCI score > 3 was also associated with more frequently administration of inadequate empirical treatment, according to previous authors [43]. Blot et al. reported that bacteremia of MDR *Pseudomonas* spp. was a risk factor for mortality [39]. This finding is in contrast with our results, in which the isolation of species other than *A. baumanii* and *K. pneumoniae* was associated with better survival.

One-third of MDR GNB BSIs in our cohort were reported among COVID-19 patients who were currently hospitalized during the study period, which highlights the significance of severe secondary infections in co-infected patients during the remission phase of the pandemic. Indeed, COVID-19 infection modified the incidence and severity of nosocomial infections in several countries [50]. In recently published data from our hospital, an increase in secondary BSIs among COVID-19 patients [29], which was in alignment with other reports [51]. In 2020, we observed a notable increase in BSIs presenting with more resistant phenotypes of the isolates when compared with the respective rates before the onset of the pandemic. A remarkable increase (almost 50%) of *K. pneumoniae* carbapenem resistance rates was observed between 2019 and 2020. Resistance to colistin also increased for *A. baumannii*, *K. pneumoniae*, and *P. aeruginosa*, the three more common endemic species in Greek hospitals. Notably, the incidence of BSIs in COVID-19 patients in our hospital during the second epidemic wave was one of the highest published in the literature, whereas the more prevalent causative pathogens were MDR Gram-negative [29]. This observation could be possibly explained by the prolongation of hospitalization and the extensive antimicrobial regime that these patients received. However, COVID-19 co-infection was not a risk factor for mortality in our study during the remission phase of the pandemic, as reported here.

Furthermore, we studied whether time for treatment modification played a role in mortality, as we assessed actions such as the discontinuation of redundant antibiotic agent and if the number of active agents of both empirical and targeted treatment was associated with the main outcome of death on day 28. Even though it would be expected that more effective antimicrobials in a prescribed regimen would be life-saving for hospitalized patients, our data did not confirm an association with lower 28-day mortality. Additionally, modification of treatment post-susceptibility results and discontinuation of unnecessary agents had no impact on the main outcome of interest.

Previous studies reported that inadequate treatment is associated with higher odds of negative outcomes [52]. In areas of widespread resistance to broad-spectrum antibiotic agents, the implementation of molecular rapid diagnostic tests may be a key for prompt adequate antimicrobial therapy [53,54]. In this study, we also evaluated the possible effect of using RDT on mortality in patients with MDR GNB bacteremia. Mohayya et al. recently showed that reduction in the duration of inadequate empirical treatment was associated with better outcomes and although not statistically significant, the finding was notable and may favor the use of RDT as a useful tool for adequate targeted treatment in the context of antimicrobial resistance strategies [55]. Although the implementation of antibiotic stewardship protocols and the progress and handy release of diagnostic tools might optimize appropriate empirical therapy, selecting appropriate empirical therapy remains a challenge, particularly for resistant pathogens. Recently published data from a large cohort in the US display a positive effect of appropriate empirical treatment on mortality during hospitalization [56].

More recent studies assessed the impact of rapid diagnostics in outcomes of patients with MDR infections [57,58]. These studies demonstrated an improvement in the administration of prompt adequate treatment using RDTs, findings consistent with our data, which report an improvement in time of first modification. Other authors reported improved exploitation of antibiotics for Gram-negative bacteria in critically ill patients. Our data add to the literature by expanding these findings to all patients with MDR Gram-negative bacteremia, not just ICU patients, suggesting a broader real-world encounter. Furthermore, our study was conducted during the remission year of COVID-19 pandemic, so the texture of the cohort was miscellaneous regarding comorbidities and disease severity.

Advances in diagnostic approaches, as well as the implementation of antimicrobial stewardship programs, may play an important role in ensuring that patients receive adequate treatment in a timelier fashion than in the past [59,60,61]. However, data are conflicting regarding the impact of RDT use on clinical outcomes with either optimization or no impact on clinical outcomes [58,62,63,64]. Babowicz et al. suggested improvement in mortality rates in contrast with our results and previous large studies [62]. In this study, the use of molecular rapid diagnostic tests did not seem to have a positive effect on reduction in the mortality rate among patients with MDR GNB bloodstream infections. Despite not improving the outcome of interest, rapid modification and augmentation of adequate treatment rates may potentially upgrade long-term outcomes.

Despite the fact that the concomitant isolation of GNB generally is not reported to affect mortality in patients with MDR *A. baumanii*, it was associated with worst outcomes in general [65]. In this study, monomicrobial infections were not associated with lower mortality rates from all MDR Gram-negative bacteria, which is also an important finding and compatible with other authors. Although multiple studies have reported higher mortality rates in bacteremic patients with polymicrobial infection [66,67], the attributable mortality rate varied depending on the causative pathogen isolated [68,69]. Compared with monomicrobial bacteremia, polymicrobial bacteremia of P. aeruginosa [70] was associated with higher mortality, while polymicrobial *K. pneumoniae* bacteremia did not led to poorer outcomes [71]. These findings indicate that the influence of polymicrobial bacteremia on prognosis should be assessed separately, as we tried to highlight further in this study.

### Limitations

This study has few limitations. First of all, being a single-center study, the results might have been affected by the practices exclusively applied in this particular healthcare facility, thereby limiting the generalizability of our findings. However, the medical wards and the ICU are part of the large tertiary care hospital that serves patients from different regions of Northern Greece. Secondly, no antibiotic dosing data and modification of dosage administration were available in this study, so it is not fully confirmed that patients received optimal treatment, a factor that might have an impact on outcome. This uncertainty might have affected the definition of adequate antibiotic therapy. Third, due to the study’s retrospective design, it may not account for all confounding factors. However, precise consideration was taken to minimize these factors. Nevertheless, we attempted to collect all study-related information for all patients. By including merely Gram-negative bacteremic patients, we tried to mitigate the risk of including contaminated blood cultures, which did not require treatment. Additionally, MDR GNB infections remain a major issue in Greek hospitals. The strength of the study could be impacted by not assessing risk factors for the development of MDR GNB BSIs in order to suggest effective measures for prevention of difficult to treat nosocomial infections. Lastly, the small size of the cohort limited the ability to perform multivariate analysis separately for *A. baumanii* and *K. pneumoniae* BSI cases, to further investigate impact of the responsive bacteria on mortality. Despite the limitations, the study has several strengths. The study focused on MDR GNB bloodstream infections on both wards and ICU and reported on important clinical outcomes, such as mortality rate, which remains high in an endemic area, but literature data are still inconsistent. In addition, a detailed description on the use of antibiotics and actions taken within the first 24 h of susceptibility results release were also presented, in a setting where rapid diagnostic tests are in use for infection control purposes. The greatest strength of our study is its real-world impact assessment, which might set a guide for improving clinical outcomes in patients with difficult to treat bacteremias and reinforce nosocomial infection prevention practices for clinical management.

## 5. Conclusions

In conclusion, this study aimed to assess clinical prognosis through 28-day all-cause mortality among hospitalized patients with bloodstream infections of multidrug-resistant Gram-negative bacteria. We report a rather high mortality rate in our cohort, which derives from an endemic region for MDR GNB unlike other studies. The severity of bacterial infections, indirectly assessed by higher PCT count, was an independent predictor of mortality, regardless other risk factors, which is consistent with previous studies and highlights its use in daily practice.

Several factors that could affect the outcome of interest were investigated in this study in both ICU and ward settings; nevertheless, it did not lead to conclusive results. Data on adequate empirical treatment, modifications upon susceptibility results, and de-escalation of antimicrobials were not associated with better survival. Overall, our study has revealed high rates of MDR Gram-negative BSI episodes among the hospitalized COVID-19 patients, a finding with significant implications for active surveillance and the need for clinical management with appropriate antibiotics for secondary infections even during a remission phase of the pandemic. Finally, the use of molecular rapid diagnostic tests did not seem to have a positive effect on the reduction in the mortality rate among patients with MDR GNB bloodstream infections. The retrospective single-center nature of the study could have a negative impact on the strength of the study and limits the generalizability of the findings. A judicious selection of a broad empirical antimicrobial regimen is essential, but a comprehensive approach would also be warranted to further improve outcomes. In summary, further prospective studies are needed to define optimal strategies for adequate empirical treatment and management in endemic for MDR Gram-negative bacteria regions.

## Figures and Tables

**Table 1:** Baseline characteristics of patients with MDR GNB bloodstream infections.

Variable	Total (*n* = 157)	28-Day Mortality	
		Alive (*n* = 78)	Dead (*n* = 79)
Age, mean (sd)	67.63 (14.14)	62.98 (14.33)	72.28 (12.40)
Ward, *n* (%) ICU Other	66 (42.04) 91 (57.96)	29 (37.18) 49 (62.82)	37 (46.84) 42 (53.16)
Creatinine Baseline, *n* (%) <1.2 mg/dL 1.2–1.9 mg/dL 2–3.4 mg/dL 3.5–4.9 mg/dL ≥5 mg/dL	123 (78.21) 25 (16.03) 6 (3.85) - 3 (1.92)	65 (84.42) 8 (10.83) 3 (3.85) - 1 (1.30)	57 (72.15) 17 (21.52) 3 (3.38) - 2 (2.53)
Charlson Comorbidity Index mean (sd)	3.89 (2.02)	3.17 (1.82)	4. 61 (1.96)
ICU days, median (IQR)	1 (0, 30)	3 (0, 45)	1 (0, 18)
Hospital days, median (IQR)	30.5 (19, 55)	47 (25, 77)	24 (16, 38)
PCT, *n* (%) <1 ≥1	54 (44.26) 68 (55.74)	36 (46.15) 24 (30.77)	18 (22.78) 44 (55.70)
Time to adequate antimicrobial therapy ≤24 h >24 h None	49 (31.41) 33 (21.15) 74 (47.44)	29 (37.18) 17 (21.79) 32 (41.03)	20 (25.36) 16 (20.25) 42 (53.16)
GNB, *n* (%) *A. baumanii* *K. pneumoniae* Other species	75 (49.02) 39 (25.49) 39 (25.49)	31 (39.74) 19 (24.36) 26 (33.33)	44 (55.70) 20 (25.32) 13 (16.46)
Type of bacteremia, *n* (%) Primary Secondary	93 (59.24) 64 (40.76)	48 (61.54) 30 (38.46)	45 (56.96) 34 (43.04)
Number of pathogens isolated, *n* (%) 1 ≥2	135 (86.54) 21 (13.46)	68 (87.18) 10 (12.82)	67 (84.81) 11 (13.92)
FilmArray use, *n* (%) No Yes	9 (5.73) 148 (94.27)	3 (3.85) 75 (96.15)	6 (7.59) 73 (92.41)
Active antibiotics in empirical treatment, *n* (%) 0 ≥1	108 (69.23) 48 (30.77)	49 (62.82) 29 (37.18)	59 (74.68) 19 (24.05)
Active antibiotics in targeted treatment, *n* (%) 0 ≥1	78 (50.00) 78 (50.00)	36 (46.15) 42 (53.85)	42 (53.16) 36 (45.56)
Intervention: Discontinuation of additional antibiotic, *n* (%) No Yes	99 (63.46) 57 (36.54)	54 (69.23) 24 (30.77)	45 (56.96) 33 (41.77)
COVID-19 co-infection, *n* (%) No Yes	104 (66.24) 53 (33.76)	50 (64.10) 28 (35.90)	54 (68.35) 25 (31.65)
MBL production	49 (31.21)	25 (32.05)	24 (30.38)
KPC production	36 (22.93)	15 (19.23)	21 (26.58)
Empirical: Colistin	22 (14.01)	12 (15.38)	10 (12.66)
Empirical: Tigecycline	17 (10.83)	4 (5.13)	13 (16.46)
Empirical: CAZ/AVI or MER/VAR	1 (0.64)	0 (0.00)	1 (1.27)
Empirical: Col + Tig	18 (11.46)	13 (16.67)	5 (6.33)
Empirical: Tig + CAZ/AVI	1 (0.64)	0 (0.00)	1 (1.27)
Empical: Tig + Col + CAZ/AVI	2 (1.27)	2 (2.56)	0 (0.00)

ICU: Intensive Care Unit; PCT: Procalcitonin; MBL: Metallo-β-Lactamases; KPC: Klebsiella-producing Carbapenemases; Tig: Tigecycline; Col: Colistin; CAZ/AVI: Ceftazidime/Avibactam; MER/VAR: Meropenem/Varborbactam; other species: *P. aeruginosa*, *P. mirabilis*, *E. cloacae*, *P. stuartii*.

**Table 2:** Univariate analysis for 28-day mortality.

Variable	28-Day Mortality	
	OR (95%)	*p*-Value
Age	1.05 (1.03, 1.08)	<0.001 *
Ward ICU Other	Ref. 0.67 (0.36,1.27)	0.221
Creatinine baseline <1.2 mg/dL 1.2–1.9 mg/dL 2–3.4 mg/dL 3.5–4.9 mg/dL ≥5 mg/dL	Ref. 2.42 (0.97, 6.05) 1.14 (0.22, 5.87) - 2.28 (0.20, 25.82)	0.057 0.875 - 0.505
Charlson Comorbidity Index	1.51 (1.25, 1.83)	<0.001 *
ICU days	0.98 (0.97, 0.99)	0.016 *
Hospital days	0.97 (0.95, 0.98)	<0.001 *
PCT <1 ≥1	Ref. 3.67 (1.73, 7.79)	0.001 *
Time to adequate antimicrobial therapy ≤24 h >24 h None	Ref. 1.36 (0.56, 3.32) 1.90 (0.92, 3.95)	0.493 0.085
GNB *A. baumanii* *K. pneumoniae* Other species	Ref. 0.74 (0.34, 1.64) 0.35 (0.16, 0.79)	0.452 0.011 *
Type of bacteremia Primary Secondary	Ref. 1.21 (0.64, 2.29)	0.560
Number of pathogens isolated 1 ≥2	Ref. 1.12 (0.44, 2.80)	0.815
FilmArray use (Y/N) No Yes	Ref. 0.49 (0.12, 2.02)	0.321
Active antibiotics in empirical treatment, *n* (%) 0 ≥1	Ref. 0.54 (0.27, 1.09)	0.085
Active antibiotics in targeted treatment, *n* (%) 0 ≥1	Ref. 0.73 (0.39, 1.38)	0.337
Intervention: Discontinuation of additional antibiotic, *n* (%) No Yes	Ref. 1.65 (0.85, 3.19)	0.136
COVID-19 co-infection, *n* (%) No Yes	Ref. 0.83 (0.74, 1.59)	0.573
MBL production No Yes	Ref. 0.93 (0.47, 1.81)	0.821
KPC production No Yes	Ref. 1.52 (0.72, 3.23)	0.275
Empirical treatment: Colistin No Yes	Ref. 0.79 (0.32, 1.97)	0.623
Empirical treatment: Tigecycline No Yes	Ref. 3.64 (1.13, 11.73)	0.030 *
Empirical treatment: Col + tig No Yes	Ref. 0.34 (0.11, 1.00)	0.050

ICU: Intensive Care Unit; PCT: Procalcitonin; GNB: Gram-negative bacteria; MBL: Metallo-β-Lactamases; KPC: Klebsiella- producing Carbapenemases; Col: Colistin, Tig: Tigecycline. Other species: *P. aeruginosa, P. mirabilis, E. cloacae, P. stuartii.* OR: Odds Ratio, CI: Confidence Interval; * Statistically significant at level 0.05.

**Table 3:** Multivariable analysis for 28-day mortality.

Variable	28-Day Mortality			
	Univariate Analysis		Multivariable Analysis	
	OR (95%)	*p*-Value	OR (95%)	*p*-Value
Age	1.05 (1.03, 1.08)	<0.001 *	1.03 (0.98, 1.08)	0.234
Ward ICU Other	Ref. 0.67 (0.36,1.27)	0.221	Ref. 0.54 (0.21, 1.42)	0.218
Charlson Comorbidity Index	1.51 (1.25, 1.83)	<0.001 *	1.25 (0.88, 1.77)	0.213
PCT <1 ≥1	Ref. 3.67 (1.73, 7.79)	0.001 *	Ref. 2.84 (1.13, 7.11)	0.025 *
Time to adequate antimicrobial therapy ≤24 h >24 h None	Ref. 1.36 (0.56, 3.32) 1.90 (0.92, 3.95)	0.493 0.085	Ref. 1.36 (0.40, 4.61) 1.45 (0.50, 4.19)	0.616 0.494
GNB *A. baumanii* *K. pneumoniae* Other species	Ref. 0.74 (0.34, 1.64) 0.35 (0.16, 0.79)	0.452 0.011 *	Ref. 0.78 (0.29, 2.12) 0.74 (0.24, 2.23)	0.635 0.588
Empirical treatment Tigecycline No Yes	Ref. 3.64 (1.13, 11.73)	0.030*	Ref. 3.66 (0.64, 21.08)	0.146
Empirical treatment Col + tig No Yes	Ref. 0.34 (0.11, 1.00)	0.050	Ref. 0.64 (0.15, 2.74)	0.544

ICU: Intensive Care Unit; PCT: Procalcitonin; GNB: Gram-negative Bacteria; Tig: Tigecycline; Col: Colistin; Other species: *P. aeruginosa*, *P. mirabilis*, *E. cloacae*, *P. stuartii.* OR: Odds Ratio, CI: Confidence Interval; * Statistically significant at level 0.05.

## Data Availability

Not applicable.
